# The Predominant Proteins that React to the MC-20 Estrogen Receptor Alpha Antibody Differ in Molecular Weight between the Mammary Gland and Uterus in the Mouse and Rat

**Published:** 2012-03

**Authors:** Aliccia Bollig-Fischer, Archana Thakur, Yuan Sun, Jiusheng Wu, D. Joshua Liao

**Affiliations:** 1 *Karmanos Cancer Institute, Wayne State University, Detroit, MI 48201, USA;*; 2 *Hormel Institute, University of Minnesota, Austin, MN 55912, USA*

**Keywords:** estrogen receptor alpha, mammary gland, breast cancer, uterus

## Abstract

There are many estrogen receptor α (ERα) antibodies available but few of them target a rodent ERα. Using the MC-20 antibody raised against the C-terminus of mouse ERα, we show in this communication that in the mammary gland of female mice and rats, the wild type (wt) ERα was detected on immunoblots as a dominant protein only during lactation, and the protein was lactating specific as it migrated slightly faster than the 67-kD wt ERα in the uterus, likely due to a different phosphorylation status. In contrast, in the nulliparous, pregnant, involuting and involuted mammary glands, the dominant protein recognized by MC-20 was about 61-kD, which is dubbed herein as “MC-20 reactive protein” or MC20RP in abbreviation as its identity is unknown. Our results showed that it was not derived from proteolysis or de-phosphorylation of the 67-kD ERα and was unlikely to be translated from an ERα mRNA variant. Ovariectomy decreased the lactating specific wt ERα but increased the 61-kD MC20RP in the mammary tumors from MMTV-c-*myc* transgenic mice but these two proteins in the uterus were unaffected. The 61-kD MC20RP was decreased in the mammary tumors, compared with proliferating mammary glands, in estrogen-treated ACI rats. These results suggest that while the lactating specific wt ERα alone or together with the MC20RP may sustain lactation, the MC20RP may support proliferation of the mammary gland and some mammary tumors.

## INTRODUCTION

In the mid 1990s, the demand for a good antibody to detect estrogen receptor α (ERα) of rodent origins was soaring, largely because this receptor plays critical roles in proliferation, differentiation, and carcinogenesis of the mammary gland and prostate (1-4), and thus triggers a dramatic increase in studies on these aspects with transgenic or knockout animal models. Moreover, it is very difficult, if not impossible, to obtain mammary tissue from pregnant or lactating women, making animal tissue an indispensable substitute for study of the role of ERα in these stages of the mammary gland.

**Figure 1:**
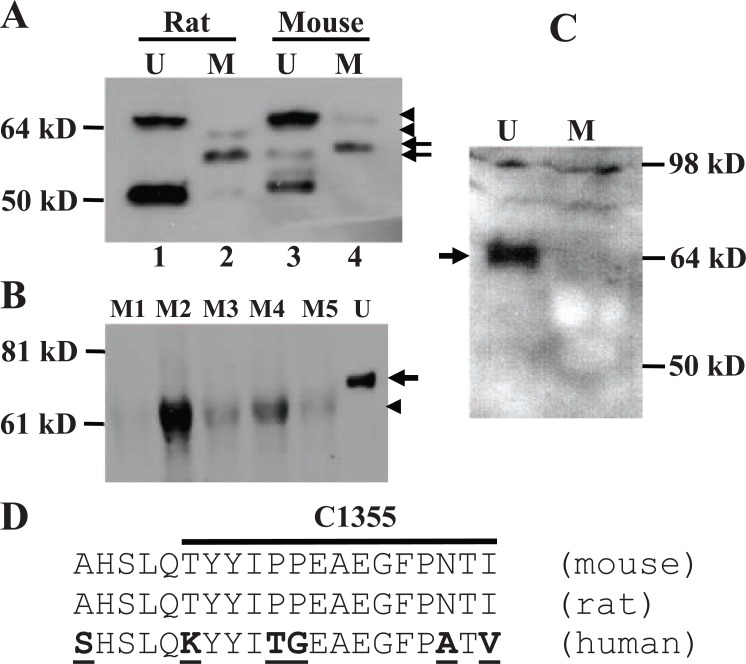
Immunoblot detection of the 67-kD wild type (wt) ERα (arrowhead) and a 61-kD protein MC20RP (arrow). A: The 67-kD wt ERα is detected at high abundance in the uterus (U) by MC-20 antibody. However, the wt ERα in the mammary tissue (M) from a virgin CD rat and a FVB mouse is only faintly detected and, in the rat, it migrated slightly faster than its counterpart in the uterus (bottom-arrowhead in lane 2 vs top-arrowhead in lane 1). In the mammary tissue, the dominant protein recognized by MC-20 is estimated as 61-kD, although it also migrates slightly slower in the mouse than in the rat (top-arrow in lane 4 vs bottom-arrow in lane 2). The second band in the mouse uterus might be the uterine 61-kD MC20RP, but it cannot be sure as it migrates slightly faster than the 61-kD MC20RP in the mouse mammary tissue. The band at about 50-kD in the mouse and rat uteri is an ERα isoform that has been well described in the literature and is not the focus herein (5, 10, 22-24). B: The 67-kD wt ERα was detected by MC20 only in the mouse uterus (U), not in the mammary tissue from five virgin female FVB mice (M1-M5). In the mammary tissue, the protein detected is smaller than that in the uterus and its abundance varies greatly among the five animals, but comparison among samples should be made with caution because virgin mammary tissue is dominated by fat-tissue and varies greatly in the amounts of other cell types. C: The Ab-17 antibody raised against the N-terminus of human ERα can recognize the wt ERα in mouse uterus (U), not the 61-kD protein in the mouse mammary tissue (M). The blot is relatively murky because a slightly excessive amount of antibody was used to ensure that its affinity to the 61-kD MC20RP is indeed poor. D: Comparison of the sequences of the last 20 amino acids of the mouse, rat and human ERα. These 20 amino acids in the mouse and rat are identical and are used as the immunogen for the MC-20 antibody, whereas the last 15 amino acids are used as the immunogen for the C1355 antibody. The human sequence used as the immunogen of the HC-20 antibody differs from that of the mouse and rat by six amino acids that are boldfaced and underlined. Figures 1A and 1C are representative of similar analyses of at least three independent samples.

The ERα encoded by esr1 gene in the human, mouse and rat has 595, 599 and 600 amino acids with calculated molecular weights of approximately 66216, 66955 and 67030 Daltons, respectively. Species differences in the ERα peptide sequences are great enough to create species specificity of antibodies. At that time the 1D5 clone of ERα antibody, which was developed with the N-terminus of the human ERα as the immunogen, was generally considered the only one good for immunohistochemical detection with paraffin embedded human tissues, although there were other antibodies reported to be immunohistochemically reactive. However, the affinity of 1D5 to the ERα of rodent origins was not satisfactory, according to Joshua Liao’s experience (Liao *et al*, unpublished data). Liao *et al* thus searched for other commercial antibodies that were developed using a rodent ERα as the immunogen. The MC-20 antibody that was newly (in 1995) provided by Santa Cruz Biotechnology Inc. (www.scbt.com) was the only one they could find and was, at that time, much more cost-effective than 1D5. The immunogen for MC-20 is the last 20 (the 580th-599th) amino acids of the mouse ERα; this sequence is identical to the C-terminus of the rat ERα but differs from the C-terminus of the human ERα by six amino acids, as illustrated in Figure 1D. In 1998 Liao *et al* reported for the first time that MC-20 has a strong affinity to the rat ERα in western blot assay and in immunohistochemical staining with paraffin-embedded tissue (5). This paper was immediately listed as a reference for this antibody in the catalogue of Santa Cruz Biotechnology Inc. Soon afterwards, this antibody widely appeared in publications on the mouse, rat and hamster ERα. It is now a commonly used antibody in studies of rodent ERα in the literature, in part because until now still there are few commercially available antibodies raised against ERα of rodent origin, particularly if antibodies that sometimes are mistakenly described to be raised by using mouse ERα are excluded, such as the 6F11 clone (6).

Liao *et al*’s original study with the MC-20 antibody determined the expression of ERα in proliferating mammary glands and tumors induced by estrogen and androgen in Noble rats (5, 7). Multiple bands were detected on immunoblots of rat uterus samples included as positive controls, mainly migrating at about 116, 98, 67, 58, 54, 46, 36 and 33 kD. Mammary tissues and tumors also expressed most of these bands but not the 67-kD wild type (wt) ERα. Instead, mammary tissue expressed a protein at about 61-kD, slightly smaller than the 67-kD ERα expressed in the uterus. Actually, this small difference in molecular weight was not realized then and thus was not discussed in that paper, in part because pre-neutralization of the antibody with synthetic immunogen peptide could not block the recognition of the 61-kD protein, suggesting that this protein is nonspecific. However, in our later practice, different batches of MC-20 antibody that is pre-neutralized with different batches of synthesized immunogen peptide sometimes could block the 61-kD protein. This inconsistency is explainable because the conformation of a synthetic peptide may be different *in vitro* and *in vivo* and because polyclonal antibodies immunized with synthetic peptides of the same sequence in different rabbits may be slightly different in epitopes, which is one of the weaknesses of polyclonal antibodies. Many western blot results published by others using the MC-20 antibody either did not provide a molecular marker or did not include a uterus sample as a positive control, making it difficult to determine whether the protein detected is really at 67-kD. Nevertheless, proteins recognized by the MC-20 antibody with molecular weights slightly smaller than 67-kD have been described in the literature, such as those dubbed as E1 (8) or ERα55 (9), ER-X (6, 10), or ERα2 (11). However, a thorough search of the literature for the ERα in the mammary gland found only a few published studies that contain immunoblot data of ERα (12-15), and none of the blots contains a uterus sample as control.

Since we are among the first ones to introduce MC-20 antibody into the community of rodent ERα research, we feel that it is important to promote the awareness of the molecular weight difference between the ERα in the mouse and rat mammary glands and the ERα in the uterus recognized by this antibody. In this communication we described three mammary-specific traits of ERα.

## MATERIALS AND METHODS

### Animals, surgery, and estrogen treatment

Virgin female CD rats and FVB mice were purchased from Taconic Farms (Germantown, NY). Virgin female ACI rats were purchased from Harlan Sprague Dawley Inc (Indianapolis, IN). The animals were housed in an AAALAC-accredited facility with water and food supplied *ad libitum*. All animal experiments including surgeries and hormone treatment were performed under institutionally approved animal protocols. Stages of mammary gland development were assigned as follows: adult virgin or nulliparous (8-12 weeks old), early to middle term pregnant (6-8 days gestation), lactating (day 7 post parturition), involuting (2-3 days post-weaning by pup removal) and involuted (two months after weaning). At these stages mice were euthanized, and mammary fat pads, uterus and other organs were quickly harvested and stored at -80°C.

Ovariectomy of mice was carried out through a small incision at the back as described previously (16). For estrogen treatment, virgin female ACI rats at 8-10 weeks of age were subcutaneously implanted through a small incision under the shoulder with a pellet of 20 mg pure diethylstilbestrol (DES) without binder as described in detail previously (5). The rats receiving DES developed frank mammary tumors 4-6 months post hormone implantation. In this period the animals were euthanized; the tumors and proliferating mammary tissues were harvested and stored at -80°C. Age-matched non-treated rats included as controls were also euthanized for collection of mammary tissue.

The breeders of MMTV-*c-myc* transgenic mice were received from the NCI, NIH and bred by us. The features of this transgenic line and the mammary tumors developed from the animals as well as the procedure of tumor tissue collection were described previously (17, 18). Four tumor-bearing animals were ovariectomized, and the tumors were harvested 10-14 days later.

### Human breast tissue

Frozen samples of uninvolved human breast tissue distant from breast tumors were provided by the Tissue Repository of Karmanos Cancer Institute at Harper University Hospital (Detroit, MI) under a protocol approved by the Human Investigation Committee of Wayne State University without disclosure of the patients’ personal information. The tissue samples were quickly frozen and stored at -80°C after surgical removal from breast cancer patients.

### Tissue lysis and *in vitro* de-phosphorylation

All animal and human tissues were homogenized with a polytron in a lysis buffer described previously (5, 17). Unless specified, the buffer contained a comprehensive list of inhibitors for proteases, kinases and phosphatases, most of which were described below in the lysis buffer used for cultured cells. For de-phosphorylation assay, mammary or uterine tissue was homogenized in a lysis buffer without phosphatase inhibitors at pH8.5. Protein lysates (250 μg total protein) were treated for 1 h at 37°C with or without (as control) calf alkaline phosphatase (325 U, Sigma-Aldrich, Inc., St. Louis, MO) as described by Asaithambi *et al* (19).

### Cell Culture and lysis

MCF-7, T47D and MDA-MB-231 (MB231) human breast cancer cell lines from ATCC were routinely grown in phenol red-free DMEM or DMEM/F12 medium (Invitrogen, Corp., Carlsbad, CA), supplemented with 10% fetal bovine serum, 50 U/ml penicillin and 50 μg/ml streptomycin. One culture of MCF7 cells contained additional 4 μg/ml insulin. The myc-MT1 cell line, developed by us from an MMTV-c-*myc* mammary adenocarcinoma, was maintained in phenol red-free DMEM supplemented with 5% calf serum. Cells at about 80% confluence were washed with 1X PBS and collected by scraping in a volume of 300 μl lysis buffer containing 50 mM Tris-HCl pH7.4, 150 mM NaCl, 1 mM EDTA pH8, 0.1% Tween-20, 10 mM β-glycerophosphate, 20 mM Na_4_P_2_O_7_, 1 mM NaF, 2 mM Na_3_VO_4_, 1 mM DTT, and 1 mM PMSF, with 1/10 volume of 10X Protease Inhibitor Cocktail (Sigma-Aldrich, Inc., St. Louis, MS) added immediately prior to use. Cells were incubated for 10 min on ice, vortexed and subsequently centrifuged. Supernatant was transferred and stored at -80°C.

### Analysis of protein degradation

Analysis of proteosome-dependent protein degradation was carried out as previously described (20). Briefly, myc-MT1 media was replaced with DMEM supplemented with 5% charcoal-stripped calf serum and maintained for 5 days, then at approximately 80% confluence. The cells were pre-treated for 1 h with 1 μM MG132 (Sigma-Aldrich, Inc., St. Louis, MO) or the vehicle (Dimethyl sulfoxide, DMSO) as non-treated control. The cells were then treated with 5 nM 17β-estradiol (E_2_), 5 μM Tamoxifen (Tam), or vehicle (ethanol) as control and, 16 h later, were harvested. Cell lysates were prepared as described above and quantified by BCA assay (Pierce Biotechnology, Inc., Rockford, IL).

### Immunoblot analysis

Discontinuous 9% or 12% SDS-PAGE was carried out. After electrophoresis, proteins were transferred to Sequi-Blot PVDF membrane (Bio-Rad Laboratories, Hercules, CA) with a blot transfer apparatus. Primary antibodies used included MC-20 rabbit polyclonal antibody (1:1000) raised against the C-terminus of mouse ERα (Santa Cruz Biotechnology, Inc, Santa Cruz, CA), HC-20 rabbit polyclonal antibody (1:1000) raised against the C-terminus of human ERα (Santa Cruz Biotechnology), and Ab-17 rabbit polyclonal antibody (1:800) raised against the N-terminus of human ERα (Lab Vision, Inc., Fremont, CA). Immunoreactive proteins were visualized by enhanced chemiluminescence using a horseradish peroxidase-conjugated donkey anti-rabbit IgG (GE Healthcare, Piscataway, NJ) at a 1:5000 dilution. For immunoblot analysis, 100 μg proteins from human cell lines and 200 μg proteins from myc-MT1 lysates were loaded into the gel. However, due to the great variance in the abundance of ERα in different tissues, the amount of proteins from different tissues and stages of mammary development varied accordingly to see comparable levels on immunoblots. Recombinant human ERα was from Sigma-Aldrich, Inc. (St. Louis, MO).

## RESULTS

### The dominant protein detected by MC-20 in the mammary gland was about 61-kD

Western blot assay showed that the MC-20 antibody detected multiple proteins in mammary tissue and uterus from adult virgin female CD rats and FVB mice (Fig. 1A). Some of these proteins, such as the abundantly expressed one at about 50 kD, have been reported to be ERα isoforms by us and others (5, 14, 15, 21). Notably, the dominant protein in the mammary gland was smaller than the 67-kD wt ERα seen in the uterus, and after more careful determination it was estimated to be about 61-kD (arrow in Fig. 1A) and is referred to as “MC-20 reactive protein” or “MC20RP” in abbreviation, since its identity is still unknown. This small difference from 67-kD to 61-kD was not manifested if the electrophoresis was not run for a long-enough time and would likely have gone unnoticed had we not included uterus samples, which in most cases did not show the 61-kD MC20RP. We are now experienced in clearly resolving the small difference in molecular weight by electrophoresis of protein samples on a 16 cm, 9% polyacrylamide gel.

In most occasions, the 67-kD wt ERα was not detectable in the mammary tissue by western blot assays, whereas the abundance of the 61-kD MC20RP varied greatly among different animals (Fig. 1B). At times the wt ERα was detected faintly in the virgin mammary tissue of some rats and mice; however, when it was detected, it sometimes migrated as its counterpart in the uterus (Fig. 1A, top-arrowhead in lanes 4 *vs* 3) but more often it migrated slightly faster than the 67-kD ERα in the side-by-side run uterus sample (Fig. 1A, bottom-arrowhead in lane 2 *vs* top-arrowhead in lane 1). This variation is mammary specific, as it had never been observed in the uterine wt ERα.

**Figure 2:**
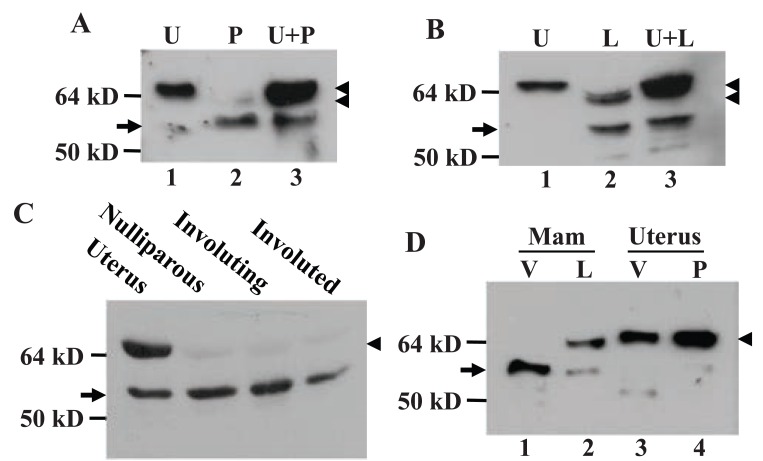
Immunoblot detection, with MC-20 antibody, of ERα in different developmental stages of the mammary gland and uterus of FVB mice. A and B: Pregnant (P) or lactating (L) mammary tissue and uterine tissue (U) were homogenized independently and in combination (U+P and U+L) at a 1:1 ratio. The uterine-derived 67-kD ERα (top-arrowhead) remains intact while the 61-kD MC20RP (arrow) remains unchanged in the U+P or U+L, relative to the level in the P or L alone. The weakly expressed wt ERα in pregnant mammary glands (bottom-arrowhead in lane 2 in A) migrates slightly faster than the wt ERα (top-arrowhead in lane 1 in A) the uterus. C: The 67-kD ERα (arrowhead) and the 61-kD MC20RP (arrow) in the uterus as well as in the nulliparous, involuting (2-day post-weaning) and involuted (two-month post-weaning) mouse mammary gland. D: Detection of the 67-kD ERα (arrowhead) and the 61-kD MC20RP (arrow) in mammary glands and uteri from virgin (V), lactating (L) or pregnant (P) mice. Figures 2A and 2B are representative of analyses of three independent samples for each stage of development. Figure 2C and 2D are representative of analyses of two independently collected tissue sets.

In the uteri from 15 virgin mice, the 67-kD wt ERα was abundantly expressed as anticipated. Several smaller proteins at or lower than 50 kD were also abundantly expressed, which were also frequently reported in other tissues or cell lines by using other antibodies (22-24) and proved by pre-neutralization with the immunizing ERα peptide (10). However, the 61-kD MC20RP was detectable in only 4 of the 15 samples, not in the majority. Even when it was detected, it was much less abundant than the 67-kD wt ERα protein (Fig. 2C), which was generally in line with our finding in the rat. Interestingly, immunoblot analysis using the Ab-17 antibody raised against an N-terminal sequence (amino acids 18-35) of human ERα detected only the 67-kD wt ERα in the mouse uterus, but not the 61-kD MC20RP in either uterus or mammary tissue even when a slightly excessive amount of antibody was used (Fig. 1C). One of the multiple possibilities is that the 61-kD MC20RP may lack part of the N-terminus. However, exhaustive RT-PCR (reverse transcription and polymerase chain reaction) and nested PCR analyses with primer pairs spanning the full-length of the transcript, from the proximal 5’untranslated region through exon 8, failed to identify a transcript that might be translated to a 61-kD protein (data not shown). Thereby the dominant 61-kD MC20RP is unlikely to arise from exon-skipping by alternative splicing.

### The 61-kD MC20RP and 67-kD ERα varied in different stages of mammary glands

Immunoblot assay using MC-20 antibody showed that the 61-kD MC20RP was the dominant protein in the nulliparous, pregnant, involuting and involuted mammary glands of the mice wherein the wt ERα was barely detected (Fig. 2A, 2C and 2D). The wt ERα started to increase in pregnant glands and its ratio to the 61-kD MC20RP reached about 1:1 (Fig. 2B) or higher (Fig. 2D) in lactating glands. However, in involuting glands two days after weaning, the abundance of the wt ERα was decreased to an undetectable level (Fig. 2C), which was similar to what was witnessed in virgin and nulliparous mammary tissue as well as in involuted glands two months after weaning (Fig. 1A, 1B and 2C). The uterus is known to undergo dramatic changes to reach physiological maturation during the transition from nulliparity to pregnancy. However, the pregnant uterus did not show any change in the expression patterns of the 61-kD MC20RP and the wt ERα (Fig. 2D). Therefore, the change in the relative abundance of the MC20RP and the 67-kD ERα seen with the development and differentiation of the mammary gland is mammary-specific and cannot be generalized to the uterus.

### There was a mammary specific isoform of the wt ERα

The wt ERα in lactating glands was sometimes present as a doublet on the immunoblot (Fig. 2B, arrow in lane 2) but in other times it appeared only as a single band (Fig. 2D, arrow in lane 2). In either case, the protein(s) always migrated slightly faster than its counterpart in the uterus (lanes 1 *vs* 2 in Fig. 2B and lanes 2 *vs* 3 in Fig. 2D). Actually, the weakly detected wt ERα in pregnant glands migrated slightly faster than the wt ERα in the uterus as well (Fig. 2A, arrowhead in lanes 2 *vs* 1), just like the faint wt ERα band seen in the virgin rat gland (Fig. 1A, arrowhead in lanes 2 *vs* 1). This form of wt ERα is denoted herein as “lactating specific isoform of the wt ERα” because it reached the highest level in lactating glands and was never observed in the uterus in our study.

### The MC20RP was not a result of proteolysis or de-phosphorylation of the 67-kD ERα

We co-homogenized uterine and mammary tissue and performed western blot assay. The results showed that in the co-homogenized protein extracts the uterine-derived 67-kD ERα was intact and the amount of the 61-kD MC20RP was not increased, leading to an unchanged ratio of the two proteins compared with what was seen in the uterus or the mammary tissue alone (Fig. 2A and 2B). Therefore, the uterine 67-kD ERα protein cannot be processed by mammary-derived factors to the 61-kD MC20RP.

**Figure 3:**
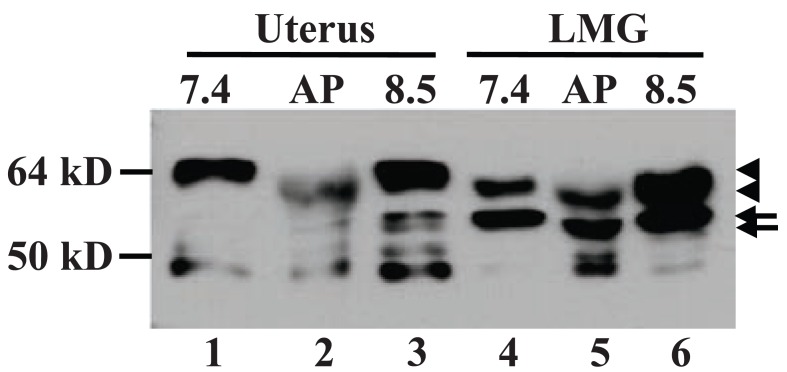
Phosphorylation status of ERα proteins. Immunoblot analysis of proteins extracted from uterus and lactating mammary glands (LMG) with lysis buffer at pH7.4 or 8.5. A portion of pH8.5 extract was incubated with calf intestine alkaline phosphatase (AP) for 1 h followed by SDS-PAGE immunoblot analysis. The higher pH condition does not change the 67-kD ERα migration (top-arrowhead in lanes 1 *vs* 3 and lanes 4 *vs* 6), but the 67-kD ERα in the AP-treated extracts from both organs is down-shifted compared with its counterpart in the non-treated extract at pH8.5 (bottom-arrowhead in lanes 2 and 5 *vs* top-arrowhead in lanes 3 and 6, respectively). Note that the AP-treated wt ERα in the uterus is down-shifted to the position of the non-treated wt (lactating specific) ERα in the LMG (bottom-arrowhead in lanes 2 *vs* 4 and 6). AP treatment also down-shifts the 61-kD MC20RP in the LMG (top-arrow in lane 6 *vs* bottom-arrow in lane 5). The experiment was run three times.

ERα can be phosphorylated at 18 or more amino acids (25, 26). To determine whether the small molecular weight difference from 67-kD to 61-kD reflect a different phosphorylation status, we prepared protein extracts of lactating mammary tissue, which expressed both proteins, and uterine tissue with lysis buffers at pH7.4 and 8.5. The pH8.5 extracts were treated with alkaline phosphatase (AP) *in vitro* that has been shown to be able to dephosphorylate ERα (19). While the higher pH condition did not change the abundance of these two proteins, AP treatment slightly down-shifted the wt ERα band in both uterus and lactating gland samples (Fig. 3, lanes 2 *vs* 3 and lanes 5 *vs* 6). AP treatment also caused an equal down-shift in the migration of the 61-kD MC20RP, but the ratio of the wt ERα to the 61-kD MC20RP remained unchanged as it was similar to that in the pH7.4 extract (Fig. 3, lanes 4 *vs* 5). These data indicate that the 61-kD MC20RP is subject to alternate phosphorylation and de-phosphorylation, but it is not derived from de-phosphorylating the 67-kD ERα. Moreover, at the pH7.4, the wt ERα in the uterine extract migrated slightly slower than its counterpart in lactating glands (Fig. 3, lanes 1 *vs* 4) as having been described in the above sections, but AP treatment down-shifted it (Fig. 3, lanes 2 *vs* 3) to the position of the wt ERα in lactating glands before AP treatment (Fig. 3, lanes 2 *vs* 4 and 6). These results suggest that the lactating specific wt ERα lacks some phosphorylation that occurs in the uterine wt ERα.

### Ovarian hormones affected expression of the MC20RP and the lactating wt ERα

**Figure 4:**
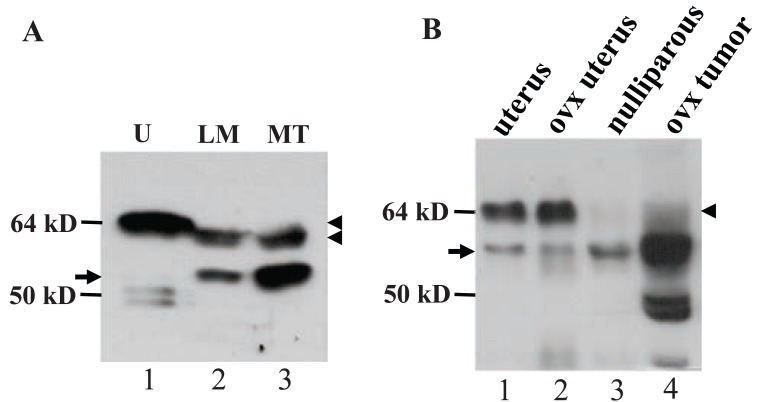
The ratio of the 67-kD wt ERα to the 61-kD MC20RP. A: The ratio of the 67-kD ERα (bottom-arrowhead) to the 61-kD MC20RP (arrow) is about 1.5:1 in lactating mammary glands (LM) but is about 1:2 in an MMTV-c-*myc* mammary tumor (MT). A uterus (U) included as a reference shows the abundantly expressed wt ERα, which is at a slightly higher molecular position than the wt ERα in the LM and MT (top-arrowhead *vs* bottom-arrowhead). B: When tumor bearing MMTV-c-*myc* mice were ovariectomized two weeks prior to the tumor collection (ovx tumors), the 67-kD wt ERα (arrowhead) level is decreased while the 61-kD MC20RP (arrow) level is increased, leading to a dramatic decrease in the ratio of the 67-kD to 61-kD proteins, compared with the ratio seen in intact animals shown in panel A. The ratio is unaffected in the uteri of ovariectomized non-transgenic mice, compared with the intact mice (lanes 1 *vs* 2), while nulliparous mammary tissue from the same ovariectomized mice shows only the 61-kD MC20RP. The figure represents analyses of tumors from three intact mice and two ovariectomized tumor bearing mice and two non-transgenic mice.

In virgin female MMTV-c-*myc* transgenic mice, the c-*myc* transgene driven by the long terminal repeat of mouse mammary tumor virus (MMTV) induces mammary adenocarcinomas (17). A surprising finding was that in these tumors, the wt ERα was the lactating specific isoform that migrated slightly faster than the wt ERα in the uterus (arrowhead in Fig. 4A, lanes 2 and 3 *vs* 1). The ratio of the wt ERα to the 61-kD MC20RP was about 1:2 in the tumors (arrowhead *vs* arrow in Fig. 4A), which was much lower than the 1:1~2:1 ratio seen in lactating glands of normal mice (arrowhead *vs* arrow in Fig. 2B, 2D and 4A) but was still much higher than the ratio in nulliparous, pregnant, involuting and involuted mammary glands of normal mice wherein the wt ERα was barely detected (Fig. 2A and 2C). However, in the mammary tumors harvested two weeks after the mice were ovariectomized, the wt ERα was decreased to a nearly undetectable level while the 61-kD MC20RP was markedly increased, leading to a dramatic increase in the ratio of the 61-kD MC20RP to the wt ERα (arrowhead *vs* arrow in lane 4 in Fig. 4B). Interestingly, ovariectomy did not change the ratio of the wt ERα to the 61-kD MC20RP in the uterus (lanes 1 *vs* 2 in Fig. 4B). Collectively, these data indicate that the levels of the 61-kD MC20RP and the wt ERα are controlled by ovarian hormones, but this control is, again, mammary-specific and does not occur in the uterus.

**Figure 5:**
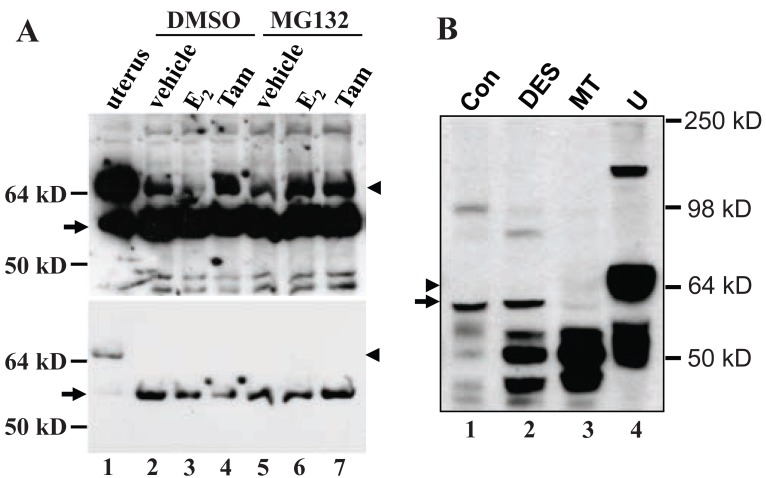
The effect of estrogen-enhanced ubiquitin-proteosome pathway on the 67-kD ERα and the 61-kD MC20RP. A: myc-MT1 cells were cultured in DMEM containing 5% charcoal-treated calf serum for 1 week prior to assay. After 1 h pre-treatment with 1 mM MG132, a proteosome inhibitor, or with DMSO vehicle, cells were treated for 16 h with 5 nM 17β-estradiol (E_2_), 5 μM tamoxifen (Tam), or ethanol (vehicle) as indicated. Cell lysates were then prepared and analyzed by immunoblot. The 61-kD MC20RP (arrow) is the only protein detected when the film was exposed for an appropriate time (bottom panel). The 67-kD ERα (arrowhead) is detected only when the film is overexposed (top panel). Note that while the 61-kD MC20RP is unaffected by MG132, E2 or tamoxifen, the 67-kD ERα is decreased by E2 in the absence of MG132 (lane 3 in the top panel). The experiment was performed twice. B: DES-induced proliferating mammary tissue (DES) or mammary tumors (MT) from female ACI rats and normal mammary tissue (Con) from non-treated rats do not express detectable amount of the 67-kD ERα (arrowhead) that is abundantly expressed in the uterus (U) from a non-treated ACI rat included as a positive control. The 61-kD MC20RP (arrow) is expressed in non-treated and DES-treated mammary tissue but is barely detectable in the tumors. Comparisons of the expression levels among different samples should be made with caution because of the great difference in the cellularity, i.e. the contents of epithelial and adipose cells, although some ERα protein isoforms with lower molecular weights may be used as internal reference.

We established a cell line from an MMTV-c-*myc* mammary adenocarcinoma, termed myc-MT1, which demonstrates a growth response upon treatment with 17β-estradiol, (E_2_, data not shown). Like the primary tumor tissue from ovariectomized animals, the myc-MT1 cells had a much greater abundance of the 61-kD MC20RP (arrow, Fig. 5A) than the wt ERα that was at such a low abundance that it could only be visualized when the immunoblot film was overexposed (Fig. 5A, arrowhead in the top panel). Treatment with E_2_ is known to induce proteosome-mediated degradation of ERα (20). Indeed, treatment of myc-MT1 cells with E_2_ decreased the wt ERα (arrowhead in Fig. 5A, lanes 2 *vs* 3 in the top panel) but pre-treatment of the cells with proteosome inhibitor MG132 prevented the E_2_-induced decrease (arrowhead in Fig. 5A, lanes 3 *vs* 6 in the top panel). However, E_2_ alone or in combination with MG132 had no obvious impact on the abundance of the dominant 61-kD MC20RP (arrow in Fig. 5A, lower panel), indicating that it is not subject to E_2_-induced, proteosome-mediated degradation in a cultured situation. Tamoxifen had no effect on degradation of the 67-kD ERα as expected and had no effect on the 61-kD MC20RP either.

DES, a potent synthetic estrogen, can induce proliferation and tumors of mammary glands in female ACI rats (27). Proliferating mammary glands from DES-treated ACI rats did not show any change in the abundance of the 61-kD MC20RP, compared with the non-treated counterpart (arrow in Fig. 5B, lanes 2 *vs* 1), although the levels of some other MC-20 reactive proteins of lower molecular weights (around 50-kD) were dramatically increased (Fig. 5B, lanes 2 *vs* 1). However, unlike what was seen in the c-*myc* transgenic mouse mammary tumor, the 61-kD MC20RP was decreased in the mammary tumors induced by DES in ACI rats (Fig. 5B, arrow in lane 3). The wt ERα was not detected in any of these mammary tissues or tumors although it was abundantly expressed in the uterus included as a positive control (Fig. 5B, arrowhead in lane 4). Comparisons of the expression levels among samples should be made with caution as the amount of adipose tissue in these samples varied greatly.

### The 61-kD protein was detected in other organs and in human breast tissue and cell lines

**Figure 6:**
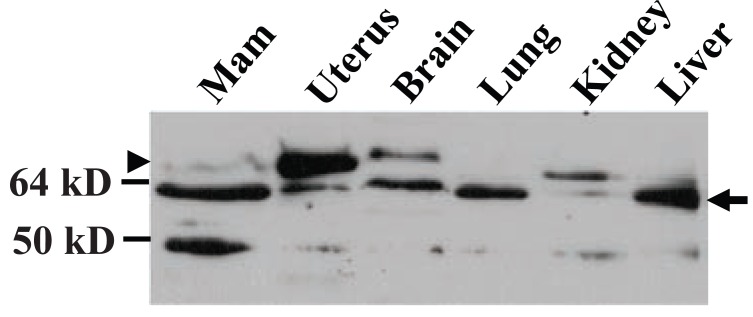
Immunoblot detection of the 61-kD MC20RP in a panel of mouse organs. These samples were resolved by a less rigorous electrophoresis (on an 8 cm, 12% acrylamide gel) than those in previous figures. The degree of separation between the 67-kD wt ERα (arrowhead; the uppermost band) and the MC20RP just beneath it (arrow) is narrowed with this more routine approach of SDS-PAGE. Note that the MC20RP is expressed in all of these tissues but the 67-kD ERα is mainly detected in the uterus, brain, kidney and mammary tissue (Mam). The figure represents analyses of two independently collected sample sets.

Western blot assay showed that MC-20 antibody detected the 61-kD MC20RP in mouse brain, lung, kidney and liver as well, although it was not always the sole or dominant protein detected (arrow in Fig. 6). The samples of these tissues were resolved by less rigorous electrophoresis (on an 8 cm, 12% acrylamide gel) than in previous figures to stress that the molecular weight difference is small from the 67-kD ERα (the uppermost band; arrowhead in Fig. 6) to the 61-kD MC20RP just beneath it (arrow in Fig. 6). The mammary sample on the western blot showed a faint band of the 67-kD ERα but a strong band of the 61-kD MC20RP, while the opposite, i.e. a much higher level of the 67-kD ERα but much lower level of the 61-kD MC20RP, were observed in the uterus sample on the same immunoblot (Fig. 6).

**Figure 7:**
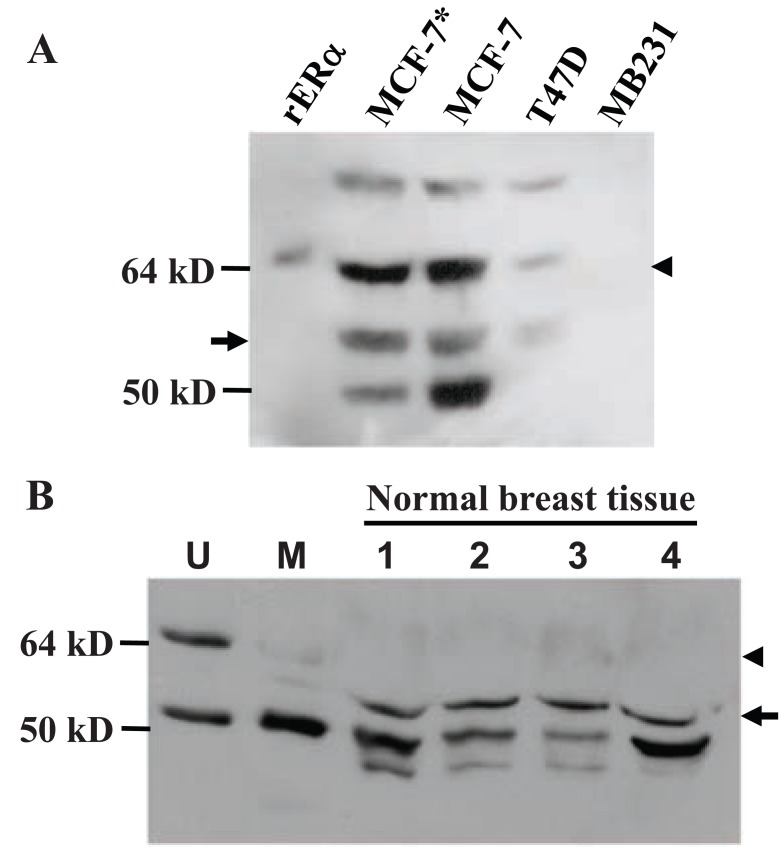
Detection of human MC20RP analog. A: Western blot assay with HC-20 ERα antibody and total lysates (100 μg proteins) from ERα positive MCF7 and T47D and negative MDA-MB231 (MB231) human breast cancer cell lines. MCF-7 cells were cultured with additional 4 μg/ml insulin or without (MCF-7*) it, as insulin may affect MCF-7 cell growth and differentiation. Purified human recombinant ERα (rERα) protein is included as a control due to the unavailability of endometrium-enriching human uterus sample. The arrow indicates a band migrating similarly to the 61-kD MC20RP, whereas arrowhead indicates the wt ERα. B: Western blot assay with HC-20 antibody and 100 μg protein lysates of uninvolved (i.e. relatively normal) breast tissue from four breast cancer patients. Uterine (U) and mammary (M) tissues from a virgin mouse are included as controls. Note that the uninvolved breast tissues show a band migrating similarly to the 61-kD MC20RP (arrow). The HC-20 antibody can detect readily the 67-kD wt ERα (arrowhead) in mouse uterus but it detects only faintly the 61-kD MC20RP and also the wt ERα in the mouse mammary tissue.

Immunoblot analysis of human breast cancer cell lines MCF7 and T47D, which are known to be ERα positive, with the HC-20 antibody raised against the C-terminus of human ERα showed that these cell lines expressed both the 67-kD wt ERα (arrowhead in Fig. 7A) and a protein estimated as the 61-kD (arrow in Fig. 7A). HC-20 antibody also detected a 61-kD protein in uninvolved breast tissue from four breast cancer patients (arrow in Fig. 7B). The HC-20 antibody could readily recognize the 67-kD wt ERα in the mouse uterus (Fig. 7B, arrowhead in lane 1) but it detected only weakly the 61-kD protein (Fig. 7B, arrow in lane 2) and the wt ERα (Fig. 7B, arrowhead in lane 2) in the mouse mammary tissue on the same immunoblot.

### ERα mRNA encompasses many downstream in-frame ATGs

We used the 20 amino acid sequence of the MC-20 immunogen (Fig. 1D) as bait to rake the entire mouse genomic sequence in the NCBI and UCSC genomic databases but did not find any peptide with significant homolog other than the ERα. We then used the mRNA sequence corresponding to the 20 amino acids as the bait to rake the databases and did not find any gene other than the *esr1*. Using other parts of the mouse *esr1* mRNA sequence as baits also failed to identify a new *esr* gene. Even the mRNA sequence of the mouse *esr2* that encodes ERβ was quite different from the *esr1* mRNA. We thus conclude that the MC20RP is not a protein product of an unidentified gene in the mouse genome.

Using DNAstar software, we analyzed the open reading frames (ORF) of the wt ERα. Since all mRNAs in the NCBI database are presented as DNA sequences, we refer AUG translation start codon and UGA stop codon as ATG and TGA, respectively. As shown in the NCBI database, the ORF of the mouse wt ERα starts with the ATG triplet at the 182-184th nucleotides (nt) of the ERα mRNA sequence (NCBI access number: NM_007956.4) and ends at the TGA of the 1979-1981st nt, encoding a protein of 599 amino acids. However, downstream of this authentic ATG, there are 25 additional ATGs that are in the same reading frame of the wt ERα and end at the 1979-1981st nt as well, thus encoding shorter proteins with truncated N-terminus but intact C-terminus (Table 1). The human ERα mRNA (NM_001122742.1) also encompasses 22 additional in-frame ATGs that are downstream of the authentic ATG triplet at the 371-373rd nt and end at the same TGA triplet as the wt ERα, i.e. at the 2156-2158th nt, thus encoding 22 shorter proteins with truncated N-terminus (Table 2). Comparison of these downstream ORFs between the mouse and the human reveals that most of them appear in both species, suggesting that they are highly evolutionally conservative (Tables 1 *vs* 2, shaded parts).

**Table 1:** Analysis of open reading frame of mouse ERα

#	Start nt	Length		#	Start nt	Length		#	Start nt	Length	
		nt	aa			nt	Aa			nt	aa

1	182th	1800	599	10	941th	1041	346	19	1262th	720	239
2	188th	1794	597	11	944th	1038	345	20	1355th	627	208
3	215th	1767	587	12	983th	999	332	21	1379th	603	200
4	278th	1704	567	13	1031th	951	316	22	1454th	528	175
5	284th	1698	565	14	1049th	933	310	23	1472th	510	169
6	518th	1464	487	15	1136th	846	281	24	1502th	480	159
7	713th	1269	422	16	1169th	813	270	25	1505th	477	158
8	719th	1263	420	17	1217th	765	254	26	1661th	321	106
9	851th	1131	377	18	1220th	762	253				

This open reading frame (ORF) analysis is based on the mouse *esr1* mRNA (NM_007956.4) sequence. “Start nt” indicates the “A” nucleotide (nt) position of the ATG triplet at the sequence. All the 26 ORFs are in the same reading frame that ends at the 1981st nt. The length of each ORF is indicated as the numbers of nt and amino acids (aa). Only ORF longer than 100 aa are shown. The shaded ones appear in the human as well.

**Table 2:** Analysis of open reading frame of human ERα

#	Start nt	Length		#	Start nt	Length		#	Start nt	Length	
		nt	aa			nt	Aa			nt	aa

1	371th	1788	595	9	1121th	1038	345	17	1532th	627	208
2	377th	1782	593	10	1160th	999	332	18	1556th	603	200
3	404th	1755	584	11	1226th	933	310	19	1631th	528	175
4	695th	1464	487	12	1259th	900	299	20	1649th	510	169
5	890th	1269	422	13	1313th	846	281	21	1679th	480	159
6	896th	1263	420	14	1394th	765	254	22	1682th	477	158
7	1028th	1131	376	15	1397th	762	253	23	1838th	321	106
8	1118th	1041	346	16	1439th	720	239				

This open reading frame (ORF) analysis is based on the human *esr1* mRNA (NM_001122742.1) sequence. “Start nt” indicates the “A” nucleotide (nt) position of the ATG triplet at the sequence. All the 23 ORFs are in the same reading frame that ends at the 2158th nt. The length of each ORF is indicated as the numbers of nt and amino acids (aa). Only ORF longer than 100 aa are shown. The shaded ones appear in the mouse as well.

## DISCUSSION

In this communication we first confirm that the MC-20 antibody has a strong affinity to the 67-kD canonical wt ERα of the rat and mouse origins, as evidenced by western blot results of uterus sample which, traditionally, was used as a positive control for the wt ERα. With this antibody ensured, we further show that in the rat and mouse mammary gland, the wt ERα is expressed as a dominant protein only in the lactation stage. In other stages of the mammary gland, including the pregnant period, the wt ERα is either undetectable or detected as a minor form, while a 61-kD MC-20 reactive protein (MC20RP) becomes dominant. Most commercial antibodies are developed using part of ERα sequence that is human specific (5), but some of these human antibodies can detect the wt and some smaller isoforms of rodent ERα. Many technical conditions can cause variations in migration of proteins and pre-stained protein markers in SDS-PAGE. Actually, such variation in molecular weights also manifests among the figures shown in this report. Therefore, we calibrate ERα proteins by loading each SDS-PAGE with not only pre-stained marker but also a uterus sample as an internal reference, which leads us to a surprising finding that ERα proteins differ between the uterus and the mammary gland in molecular weight. The observation of the 61-kD protein in the absence of the 67-kD wt ERα in a few organs raises a serious question as to whether the protein shown on published immunoblots, no matter which antibody is used, is indeed the wt ERα or rather is this 61-kD protein identity of which remains unknown. This issue needs to be considered in future studies of ERα in mammary glands with whichever antibody, since it is unclear whether many commercial antibodies can detect the 61-kD protein of rodent origin and since this protein may have its human analog as shown in Figure 7 for HC-20 antibody.

Unlike most other organs, the mammary gland differs among different ages or time periods, i.e. in embryonic stage, before puberty, after puberty, in pregnancy, in lactation, in involution and post involution. Another level of complexity is added because it differs between menopausal and postmenopausal stages and varies throughout the menstrual cycle, although this hormonal cycle is less evident in rodents that do not manifest monthly uterine bleeding. Although all these developmental or differentiation stages of the mammary gland are considered normal, lactation is the only truly normal and functional situation, considering that the gland’s function is to lactate. Only in this stage, the mammary gland expresses the highest amount of the wt ERα, leading to the highest ratio of the wt ERα to the 61-kD MC20RP, which is obviously attributed to the dominance of the glandular cells as well as to their fully-differentiated status and lactating activity, since pregnant glands that also contain a large number of epithelial cells still show a low level of this wt ERα. Another astonishing finding is that the wt ERα is mammary specific as it migrates slightly faster than its counterpart in the uterus, likely due to a different phophorylation status as de-phosphorylation of the uterine wt ERα with AP can lower its position to that of the lactating wt ERα on immunoblot (arrowhead in Fig. 3, lanes 2 *vs* 4 and 6). We name this wt ERα “lactating specific isoform” to distinguish it from the 61-kD MC20RP and to emphasize that it peaks at the lactating period and has never been observed in the uterus. We speculate that it may be this lactating specific wt ERα alone or together with the 61-kD MC20RP that supports the lactation.

We have made a few attempts to obtain the identity of the 61-kD MC20RP but all efforts failed, including an attempt to immunoprecipitate it for sequencing, due to a poor immunoprecipitation ability of MC-20 to this protein (although MC-20 can precipitate the wt ERα). Nevertheless, the abundance of MC20RP and the apparent lack of the wt ERα in pregnant mammary glands collectively suggest that it may be an ERα isoform, because it is reasonable to expect that ERα should be readily detectable during pregnancy, unless it is not a major player in sustaining proliferation and differentiation of pregnant glands, which is very unlikely (1). What remains unknown is whether it is expressed by the mammary epithelium and/or other cell types, and this cannot be determined easily by immunohistochemical staining because MC-20 antibody detects multiple proteins (5). We intend to consider glandular epithelium as its origin because its level is high in c-*myc* transgenic mammary tumors, in the myc-MT1 cell line, and in lactating mammary glands, but is low, sometimes undetectable (M1 in Fig. 1B), in the nulliparous mammary tissue that is dominated by adipose. Even if it is expressed by adipose cells or by other types of stromal cells, it can still influence glandular epithelium via a paracrine mechanism. Moreover, since this 61-kD MC20RP is also expressed in the brain, lung, kidney and liver, it probably has a more universal function, not just a role in female reproduction. Supporting this conjecture, the ERα expressed in normal human leucocytes is about 62-kD and in mouse pancreatic islet is about 58-kD, not 67-kD (28, 29).

Using MC-20 and H-184 antibodies, Kos *et al* detected a 61-kD protein in the uteri from 5 of 6 ERα knockout (αERKO) mice (8). However, we only detected the MC20RP in the uteri from 4 of 15 intact mice and did not find it reactive to the H-184 (data not shown) and the Ab-17 antibodies, both of which are raised against the N-terminus of human ERα. We had actually tried many other antibodies with different regions of human ERα as the immunogen but failed to obtain conclusive results, mostly because of poor affinity of the antibodies. Toran-Allerand *et al* have also described a 62-63 kD MC-20 reactive protein in the mouse brain, dubbed ER-X, which is expressed in αERKO mice as well (6, 10). On the other hand, the ERα2 detected by Shao *et al* with MC-20 has a molecular weight very close to the wt ERα (coined by them as ERα1), which is expressed in several tissues from intact mice but not in the uteri from αERKO mice (11). Thus, several investigators including us have described a MC-20 reactive protein in mice that is slightly smaller than 67-kD, but it is unclear whether we are describing the same protein or different proteins, since the data are partially inconsistent and none of us is able to provide the peptide sequence so far.

We also performed RT-PCR and nested PCR to analyze various ERα transcripts but found that the exon boundaries of the majority of ERα transcripts were intact. Several ERα mRNA splice variants, which were found in some human and mouse tissues and cell lines (30), were either absent or expressed at low abundance in the mammary tissue of most developmental stages (data not shown). Thus we are unable to link the 61-kD MC20RP to an alternatively spliced or initiated mRNA variant, although most other reported ERα protein isoforms can be associated with a corresponding mRNA (28, 31, 32). Moreover, the wt ERα is shown to be, as previously reported (20), targeted for estrogen-induced ubiquitination and proteosome degradation, but the 61-kD MC20RP is not. It remains to be determined whether this is the mouse analog of the ERα protein found by El *et al* in MCF7 cells that is devoid of estrogen binding (33).

All the ERα antibodies we know detect multiple bands on immunoblots (5, 10, 22-24), especially when tissues (not cell lines) were analyzed. Actually, the majority of ERα proteins expressed in human breast cancers that bind estrogens are smaller than 66-kD (22-24). The ERα expressed by many endometrial cancers is lighter than the recombinant ERα protein included as positive control on the immunoblots and is estimated as 62-kD, whereas no 66-kD wt ERα was detected (34). Among the most frequently discussed mechanisms for generating these smaller ERα proteins are mRNA variation and proteolysis (22, 35-37), but these two mechanisms cannot explain the existence of those MC-20 reactive proteins in αERKO mice (6, 8, 10), and our computational analysis fails to identify a new gene in the mouse genome that contains the MC-20 immunogen sequence. In the current gene-knockout technology, the targeted gene is knocked out by inserting a DNA fragment (usually a neo cassette) into its 5’ sequence or by conditionally deleting part of its 5’ sequence, leading to disruption of the wt ORF of the transcript and ensuing loss of the wt protein. However, usually the disrupted gene is still transcribed and in many occasions the transcript can be spliced to an mRNA that may be translated from one or more downstream in-frame ATGs. Indeed, at least two alternatively spliced mRNA variants of *esr1* are detected in αERKO mice (38-40). Therefore, translation from some downstream in-frame ATGs may be a third possible mechanism that has rarely been discussed hitherto, which may occur in αERKO (39) or conditional αERKO (41) mice as well. Generally speaking, some of the in-frame ATGs in many genes, like the 22 (in human) or 25 (in mouse) downstream in-frame ATGs of ERα, may be functional via a non-classical cap-dependent or even cap-independent mechanism of protein translation, including leaky scanning, reinitiation, IRES (internal ribosome entry site) (42-44), and CITE (cap-independent translational enhancers) (45). In the case of IRES, which seems to exist in human ERα mRNA (46), whether an in-frame ATG is functional depends in a large part on ITAFs (IRES trans-acting factor), expression of which varies among different cells or cellular situations (47, 48). Besides IRES, some relative-upstream ATGs may be used to control translation from the ATGs at their downstream (43, 44), similar to the upstream ORFs in the human ERα that regulate the ERα translation (49, 50). By using these non-classical cap-dependent or -independent translation mechanisms, cells can regulate mRNA decay or translation efficiency or can produce proteins that differ in the N-terminus and thus in some functions (42-44, 51). The possibility that the authentic ATG and some downstream ones are concomitantly functional, resulting in translation of multiple ERα protein isoforms, becomes higher because the downstream ORFs are highly conservative between the mouse and human (Tables 1 *vs* 2). Actually, the 61-kD ERα in chicken (52) and Xenopus (53) and the 45-kD ERα in human (46) may also be translated from an downstream ATG of the wt mRNA, besides from an alternatively spliced mRNA variant, even in ERKO mice (38).

The 61-kD MC20RP is highly expressed in the c-*myc* transgenic mammary tumors and can be further increased by ovariectomy. On the other hand, this protein seems decreased in DES-induced rat mammary tumors compared with the DES-induced proliferating mammary glands, although these two samples vary in adipose content. These data collectively suggest that a high level of estrogen for a long period of time may suppress this protein *in vivo*, although the suppression does not occur in myc-MT1 cells treated with E2 for a short period of time in culture. On the contrary, the lactating specific wt ERα expressed in the mammary tumors from MMTV-c-*myc* transgenic mice is decreased upon ovariectomy of the mice, suggesting that its expression required estrogen and/or progesterone. These hormonal regulations are mammary tumor specific as these two proteins in the uterus are not affected by ovariectomy.

Although voluminous studies have been published on ERα in human breast cancers, available immunoblot data is still insufficient to show whether the one expressed is the canonical uterine ERα, the lactating specific ERα, the 61-kD putative ERα, or an even smaller isoform. Likely, different breast cancers may show different ratios among different isoforms, similar to the ratio difference between the MMTV-c-*myc* tumors and the estrogen induced tumors shown herein. While blocking ERα, such as with tamoxifen, as a general strategy has been proved to be effective in treatment of roughly 50% of the breast cancers for a period of time (26, 54), we wonder whether the opposite may also work, i.e. shifting the ERα expression from some harmful, probably some smaller, isoforms to a salubrious one (probably the canonical uterine wt form or the lactating specific wt form). Therefore, it is meaningful to study the functions of different wt or smaller ERα isoforms and determine their human relevance. Unfortunately, several human uterine samples we obtained were dominated by muscle, whereas the endometrium occupies a large portion of the mouse uterus because the muscle layer is thin. While a key finding in this report is a contrast between the lactating ERα and its uterine counterpart, lack of human lactating mammary tissue and endometrium-enriching uterine sample currently hinders us from exploring whether similar molecular weight differences also appear in the human.

In summary, we observe three mammary-specific traits of ERα in the mouse and rat: 1) In most stages of the mammary gland, including the pregnant period, the dominant MC-20 reactive protein is about 61-kD, which is likely to be an ERα isoform, unless ERα is not required to sustain pregnant glands, which is less likely; 2) The wt ERα abundantly expressed in lactating mammary glands is a mammary-specific isoform that does not occur in the uterus and differs from its uterine counterpart probably by phosphorylation status; 3) The lactating wt ERα and the 61-kD putative ERα respond differently to estrogen treatment and ovariectomy from the uterine wt ERα. It is possible that the lactating specific isoform of wt ERα alone or together with the 61-kD protein sustains the lactation while the 61-kD protein supports proliferation of the mammary gland and some mammary tumors.
